# NVP-2, in combination with Orlistat, represents a promising therapeutic strategy for acute myeloid leukemia

**DOI:** 10.1080/15384047.2025.2450859

**Published:** 2025-01-12

**Authors:** Qing Zhu, Jia Cheng, Yuqing Gao, Zimu Zhang, Jian Pan, Xin Su, Danhong Fei, Linbo Cai, Juanjuan Yu, Yanling Chen, Wanyan Jiao, Di Wu, Xiaolu Li, Peifang Xiao

**Affiliations:** a Children’s Hospital of Soochow University, Suzhou, China; b Department of Pediatrics, The Third People’s Hospital of Kunshan, Suzhou, China; c Institute of Pediatric Research, Children’s Hospital of Soochow University, Suzhou, China; d Department of Hematology, Children’s Hospital of Soochow University, Suzhou, China; e Department of Pediatrics, Municipal Hospital Affiliated to Taizhou University, Taizhou, China; f Department of Pediatrics, The Third People’s Hospital of Yancheng, Yancheng, China

**Keywords:** Acute myeloid leukemia, apoptosis, CDK9, cell proliferation, c-Myc, FASN, molecular targeted therapy, SREBF1

## Abstract

Cell cycle dysregulation and the corresponding metabolic reprogramming play significant roles in tumor development and progression. CDK9, a kinase that regulates gene transcription and cell cycle, also induces oncogene transcription and abnormal cell cycle in AML cells. The function of CDK9 for gene regulation in AML cells requires further exploration. In this study, we knocked down the CDK9 to investigate its effects on the growth and survival of AML cells. Through RNA-seq analysis, we identified that in U937 cells CDK9 regulates numerous genes involved in proliferation and apoptosis, including mTOR, SREBF1, and Bcl-2. Furthermore, our results demonstrated that both CDK9 and FASN are crucial for the proliferation and survival of Kasumi-1 and U937 cells. Mechanistically, MCL1, c-Myc, and Akt/mTOR/SREBF1 may be critical factors and pathways in the combined therapy of NVP-2 and Orlistat. In summary, our study revealed that CDK9 and FASN are vital for maintaining AML cell survival and proliferation. Treatment with NVP-2 and Orlistat may be a promising clinical candidate for patients with AML.

## Introduction

Acute myeloid leukemia (AML) is a hematologic malignancy characterized by abnormal clonal proliferation of myeloid progenitors. The improvement of AML therapeutic effect is primarily attributed to the advancements in supportive care and Hematopoietic Stem Cell Transplantation (HSCT) over the past two decades. The overall 5-year survival rate of pediatric AML was around 68%.^1^ However, many patients still fail to achieve a complete remission or experience relapse after initial remission. Additionally, some patients succumb to the side effects of chemotherapy and HSCT.^2^ Research on targeted therapies for AML can provide valuable information on disease pathogenesis and progression, as well as offer potential therapeutic targets for enhancing treatment response, reducing relapse rates, and improving patients’ long-term survival and life quality.

Cyclin-dependent kinase 9 (CDK9) is a serine/threonine kinase that plays a crucial role in regulating cell cycle and gene transcription.^3^ Its activity is dependent on its association with cyclin T,^4^ controlling transcription elongation by phosphorylating the C-terminal domain of RNA Pol II.^4,5^ In addition to its role in transcription, CDK9 is also participated in cell cycle regulation,^6^ chromatin modification, and mRNA processing.^7^ Previous studies have indicated that CDK9 upregulates A-FABP, promotes the differentiation of 3T3-L1 cells, and enhances adipogenesis.^8^ It has also been shown to decrease free fatty acid levels.^9^ Furthermore, CDK9 is associated with activation of mitochondrial oxidative phosphorylation and lipid metabolism.^10^ Lipid metabolism plays a critical role in tumors, diabetes, and neurodegenerative diseases. Notably, abnormal lipid metabolism in leukemia cells has garnered increasing attention from researchers.

Metabolic reprogramming means cellular metabolic alterations to adapt to diverse pressures.^11^ In the context of cancer, cells utilize metabolic reprogramming to address nutritional alterations and microenvironmental hypoxia, thereby promoting cell survival and proliferation.^12^ Numerous studies have highlighted significant lipid metabolic reprogramming in AML cells.^13,14^ The dysregulation of the cell cycle and its corresponding metabolic reprogramming are essential in tumorigenesis. However, the specific role of CDK9 in regulating lipid metabolism genes in AML cells is still under study.

This research has identified the role of CDK9 in AML cells and observed its regulation of numerous gene expressions, such as SREBF1, in U937 cells. Our findings demonstrate that, in AML cells, treatment with the CDK9 inhibitor NVP-2 and the FASN inhibitor Orlistat resulted in proliferation inhibition and promotion of apoptosis. These results suggest a potential new therapeutic approach for AML.

## Results

### High expression of CDK9 is associated with poor prognosis in AML patients

**Figure 1.**
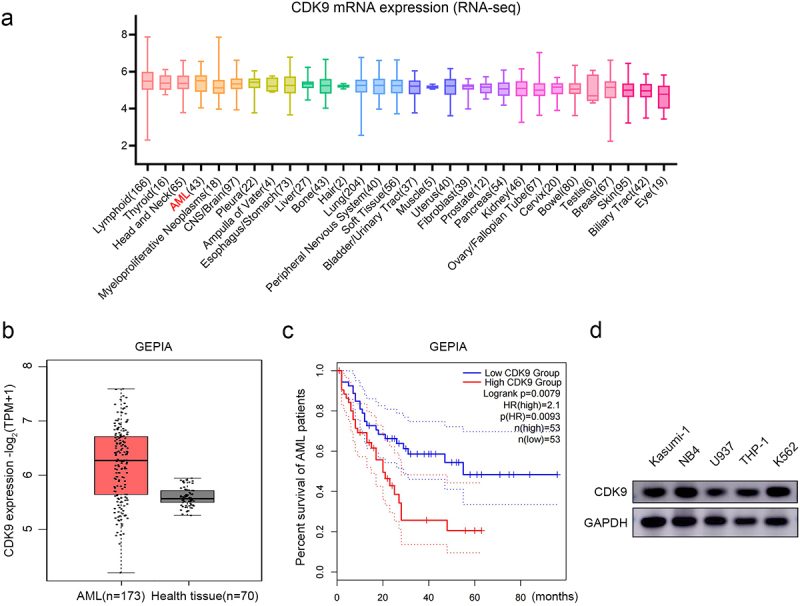
CDK9 was widely expressed in AML cells and related to prognosis. (a) Levels of CDK9 expression in common cancer cells in the CCLE database. (b) Expression of CDK9 in AML cells and normal tissues obtained from the GEPIA database. (c) Kaplan-Meier curves of CDK9 in the GEPIA database. The survival rate was markedly lower in AML patients with high expression levels of CDK9 than in those with low expression levels. (d) Western blot analyses showing the expression of CDK9 in AML cell lines, GAPDH is used as the loading control.

To investigate the functions of CDK9 in AML, we utilized two public databases: CCLE (https://sites.broadinstitute.org/ccle/.) and GEPIA (http://gepia.cancer-pku.cn/). Initially, we compared the expression of CDK9 in 30 types of tumors in the CCLE database. This finding indicated a high expression of CDK9 across all tumor cells, suggesting a potential association between CDK9 function and transcriptional activity in tumor cells (Figure 1a). Subsequently, analysis using the GEPIA database revealed no difference in CDK9 levels between AML cells and healthy tissues (Figure 1b). Furthermore, Kaplan-Meier survival plot analysis demonstrated that highly expressed CDK9 was correlated with poor prognosis for overall survival time among AML patients (Figure 1c). Additionally, Western blot analysis confirmed universal expression of CDK9 across various AML cell lines, including Kasumi-1, NB4, U937, THP-1, and K562 cell lines (Figure 1d). In summary, our findings suggest that elevated levels of CDK9 are universally present in AML cell lines and are associated with unfavorable outcomes for patients with AML.

### CDK9 knockdown suppressed AML cell proliferation and induced apoptosis

**Figure 2.**
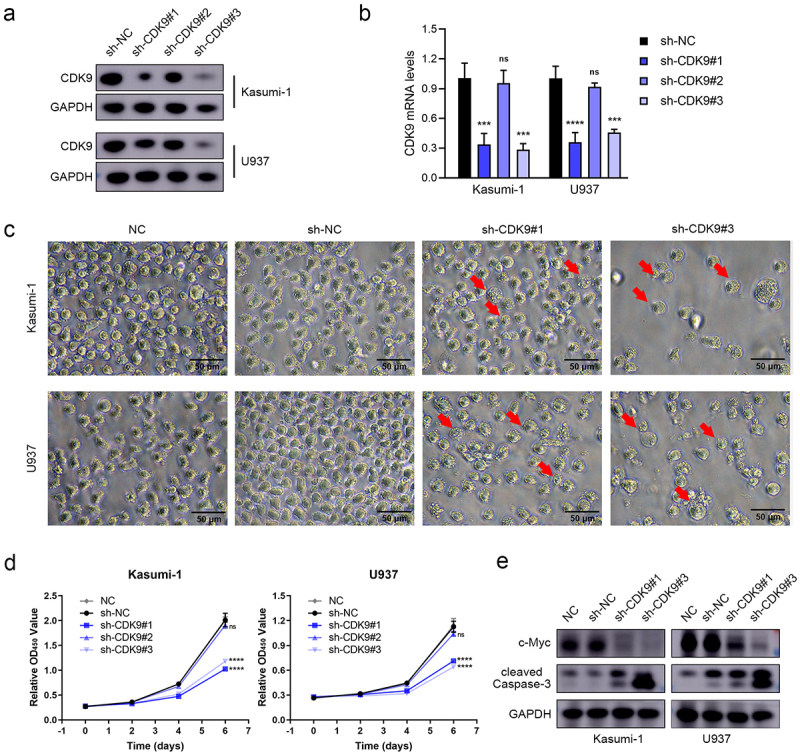
CDK9 knockdown inhibited AML cell proliferation and induced apoptosis *in vitro*. (a) Western blot analyses showing the knockdown efficiency of CDK9 in Kasumi-1 and U937 cells. (b) Knockdown efficiency of CDK9 was evaluated in Kasumi-1 and U937 cells by RT-PCR. (c) Typical apoptotic morphological changes in Kasumi-1 and U937 cells as observed by microscope. (d) Cell viability of Kasumi-1 and U937 cells infected with either a control or CDK9-directed shRNAs. (e) Western blot for cleaved caspase-3, c-Myc, and GAPDH (loading control) after CDK9 knockdown. Data are shown as mean ± SD. ns *P*  > 0.05, ***p < .001, ****P  < 0.0001.

To investigate the necessity of CDK9 for leukemia maintenance *in vitro*, shRNA systems were designed to target CKD9 expression in Kasumi-1 and U937 cells. The results demonstrated a reduction in CDK9 expression in the sh-CDK9#1 and sh-CDK9#3 groups (Figure 2a-b). Knockdown of CDK9 inhibited cell growth and induced typical apoptotic morphological changes in these AML cells compared to the controls (Figure 2c-d). Additionally, CDK9 knockdown led to decreased expressions of c-Myc and an increase in cleaved Caspase-3 expression (Figure 2e). Overall, the results implied that CDK9 is a major player in AML progression, making it a prospective therapy goal for AML.

### CDK9 knockdown demonstrated a significant antitumor effect in the leukemia mouse model

**Figure 3.**
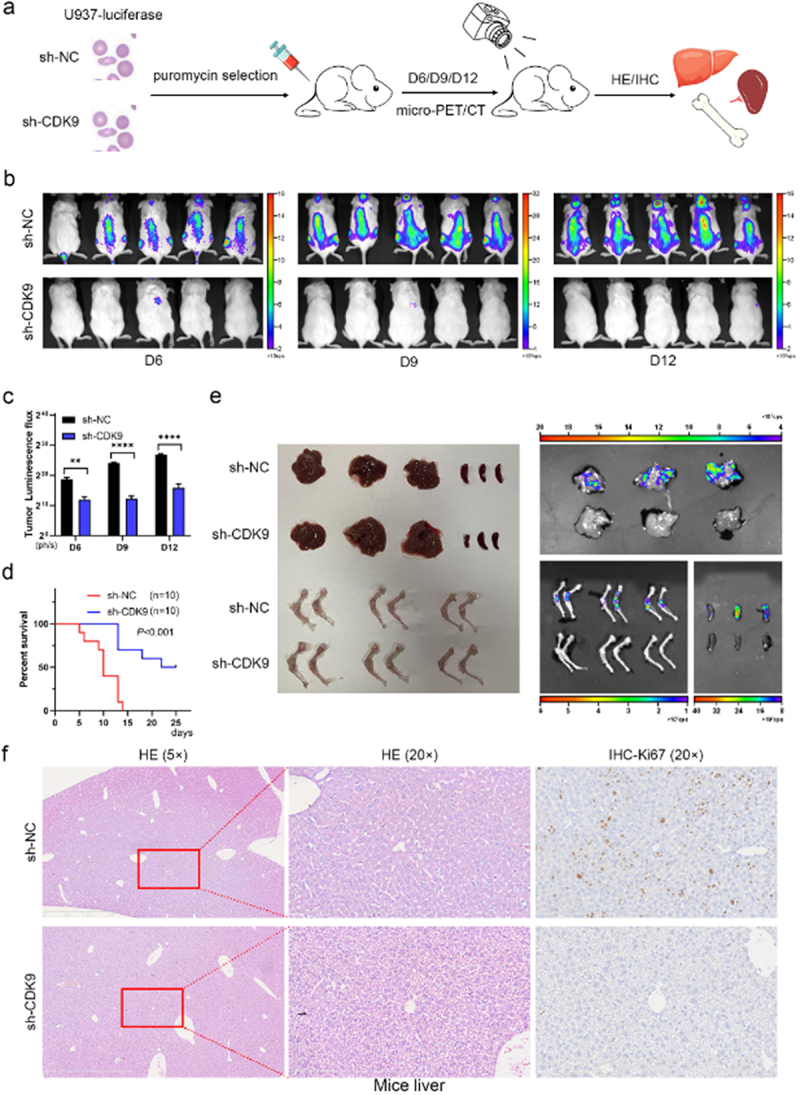
CDK9 knockdown possessed an antitumor effect in the leukemia mouse model. (a) Schematic design of the experiment. (b) Mice were transplanted with sh-CDK9 U937-luciferase cells or sh-nc U937-luciferase cells, taking representative bioluminescence images at Day 6, 9, and 12 of treatment. (c) The luminescence signal value of sh-CDK9 group is lower than that of the control group. (d) Mice transplanted with sh-CDK9 U937-Luc cells had a prolonged survival time compared with the controls. (e) Images of liver, spleen, and bone marrow from mice. (f) Representative images of mice liver by HE staining or IHC staining (Ki67). Data are shown as mean ± SD. **p < .01, ****p < .0001.

Subsequently, we investigated the *in vivo* tumorigenesis influence of CDK9 knockdown on AML cells. Specifically, M-NSG mice were injected with sh-CDK9 U937-Luc cells or sh-NC U937-Luc cells and then divided into two groups. The role of CDK9 in tumor growth was assessed through mice survival time, *in vivo* imaging, and immunohistochemistry (IHC) (Figure 3a). It showed reduced tumor bioluminescence signals in the CDK9 knockdown group on day 6, day 9, and day 12 after transplantation (Figure 3b-c). The cancer cell load among mice in the CDK9 knockdown group increased slightly but maintained a significant survival advantage (Figure 3d). *In vitro* bioluminescence imaging showed that the liver, bone marrow, and spleen were the target tissues where U937-Luc cells had implanted (Figure 3e). The results of HE staining demonstrated that U937-Luc cells invaded the liver, bone marrow, and spleen. Ki67 IHC staining confirmed that CDK9 knockdown inhibited the growth of U937-Luc cells in these tissues (Figure 3f and S1a-S1b). In summary, the results from our *in vivo* experiments were consistent with those from our *in vitro* experiments, both showing that CDK9 knockdown had an antitumor effect on leukemia.

### In U937 cell line, CDK9 regulated numerous genes, some of which were related to proliferation and apoptosis

**Figure 4.**
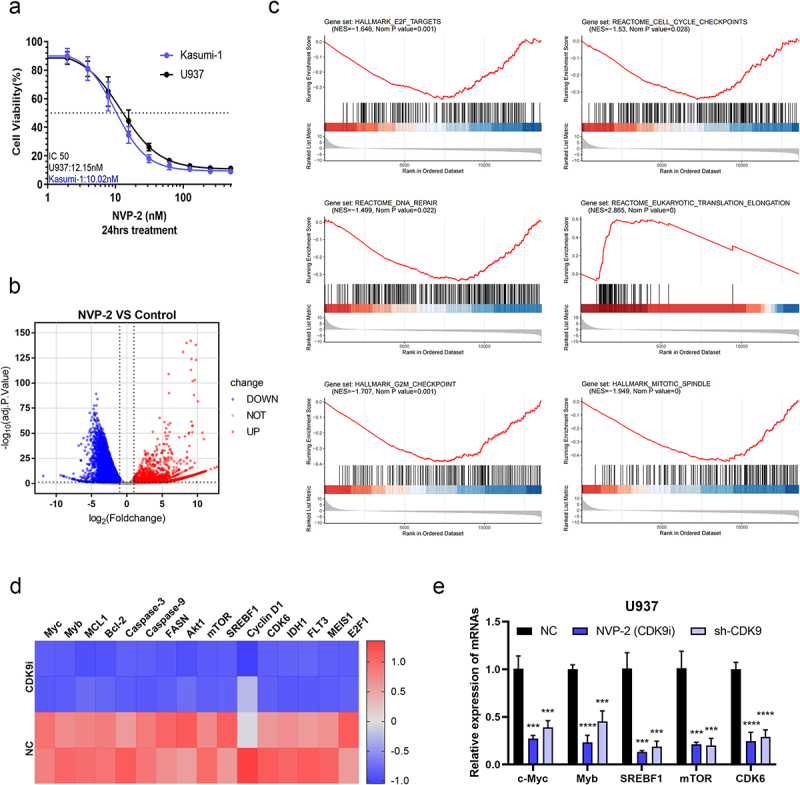
CDK9 regulated numerous genes in U937 cells. (a) Cell viability of Kasumi-1 and U937 cells treated with increasing concentrations of NVP-2 for 24 hours. (b) Volcano plots of RNA-seq showing genes down-regulated (left, blue) and genes up-regulated (right, red) in U937 cells treated with or without NVP-2 for 12 hours. (c) GSEA plots showed enrichment of the mitotic spindle, E2F, G2M, cell cycle, DNA repair, and eukaryotic translation elongation in U937 cells following CDK9 inhibition. (d) Changes in some genes associated with proliferation and apoptosis were observed upon CDK9 inhibition in U937 cells, such as the down-regulation of mTOR, SREBF1, and CDK6. (e) RT-pcr revealed the down-regulation of c-Myc, Myb, SREBF1, mTOR, and CDK6 after suppressing CDK9 with NVP-2 or knocking down CDK9 in U937 cells. Data are shown as mean ± SD. ***p < .001, ****p < .0001.

To assess the dependency of AML cell maintenance, we subsequently investigated the impact of the CDK9 inhibitor NVP-2 on AML cells. Our findings revealed that NVP-2 inhibited Kasumi-1 and U937 cell viability *in vitro* in a dose-dependent manner (24-hour IC50 for Kasumi-1: 10.02 nM, 24-hour IC50 for U937: 12.15 nM) (Figure 4a). Furthermore, we conducted the RNA-seq assay to examine the genes regulated by CDK9 in U937 cells treated with or without NVP-2 for 12 hours. Among 17,841 genes analyzed, we identified 9668 differentially expressed genes following NVP-2 treatment (Figure 4b). Gene signature enrichment analysis (GSEA) revealed that the inhibition of CDK9 affects cell cycle and apoptosis factors, including mitotic spindle, E2F, G2M, DNA repair, and eukaryotic translation elongation (Figure 4c). We observed that it also results in altered gene expression, such as reduced levels of mTOR, SREBF1, and CDK6 (Figure 4d-e). These findings underscored the vital position of CDK9 within the maintenance of AML cell survival and suggested that combination therapy targeting specific targets and pathways may enhance the efficacy of CDK9 inhibitors.

### Orlistat enhanced NVP-2 inhibitory effect in the treatment of AML cell lines

**Figure 5.**
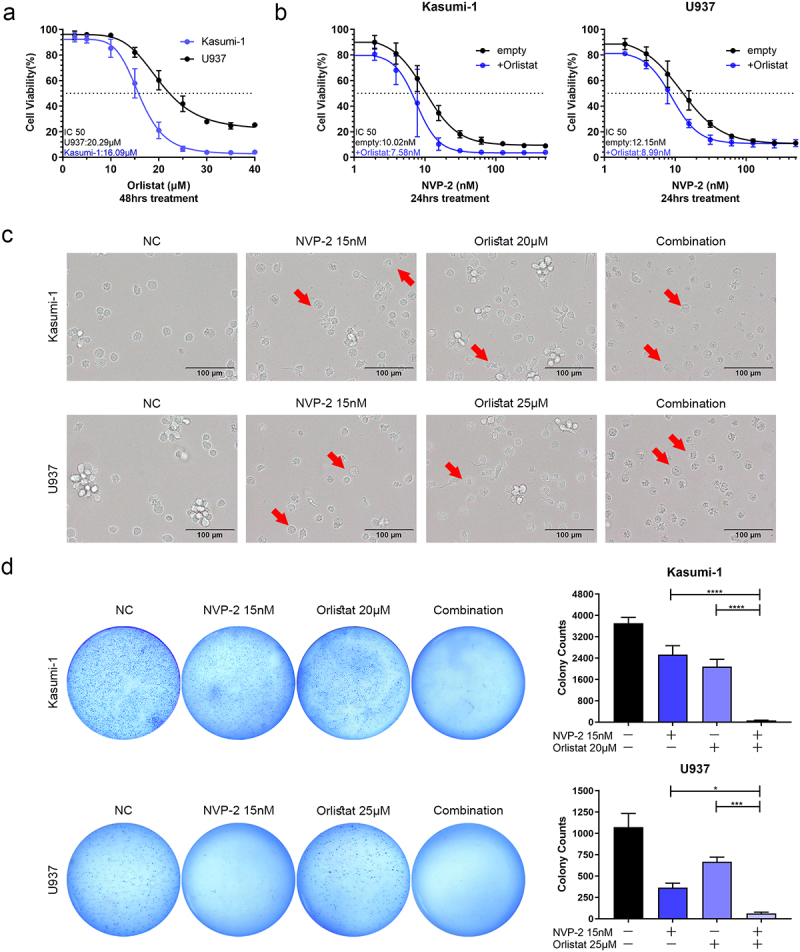
Orlistat enhanced NVP-2 inhibitory effect in the treatment of AML cell lines *in vitro*. (a) Cell viability of Kasumi-1 and U937 cells treated with increasing concentrations of Orlistat for 48 hours. (b) Cell viability of Kasumi-1 and U937 cells treated with increasing concentrations of NVP-2 combined with 10 μM Orlistat or not for 24 hours. (c) Typical apoptotic morphological changes in Kasumi-1 and U937 cells treated with NVP-2, Orlistat, or the combination as observed by microscope. (d) Colony-forming assay for Kasumi-1 and U937 cells treated with NVP-2, Orlistat, or the combination. Data are shown as mean ± SD. *p < .05, ***p < .001, ****p < .0001.

To enhance suppression, we explored combining NVP-2 with the FASN inhibitor, Orlistat. Initially, our results demonstrated that Orlistat dose-dependently inhibited Kasumi-1 and U937 cell viability *in vitro* (with a 48-hour IC50 of 16.09 μM for Kasumi-1 and 20.29 μM for U937) (Figure 5a). Subsequently, we investigated the inhibitory effect on Kasumi-1 and U937 cells after exposure to gradient concentrations of NVP-2 combined with 10 μM Orlistat for 24 hours. At equivalent concentrations of NVP-2, AML cell viability dropped in the combination group. The 24-hour IC50 value for NVP-2 in the combination group was found to be 7.58 nM in Kasumi-1 cells and 8.99 nM in U937 cells, which was lower than observed in the NVP-2 single group (Figure 5b). Microphotograph and Soft agar assay results further confirmed that the combination of NVP-2 and Orlistat significantly inhibited AML cell proliferation (Figure 5c-d and S2a). Altogether, the above results showed that Orlistat enhanced NVP-2 suppression, suggesting that a combination therapy involving both agents may represent a novel approach for treating AML.

### NVP-2 combined with Orlistat increased the anti-proliferative and apoptotic effects on AML cell lines

**Figure 6.**
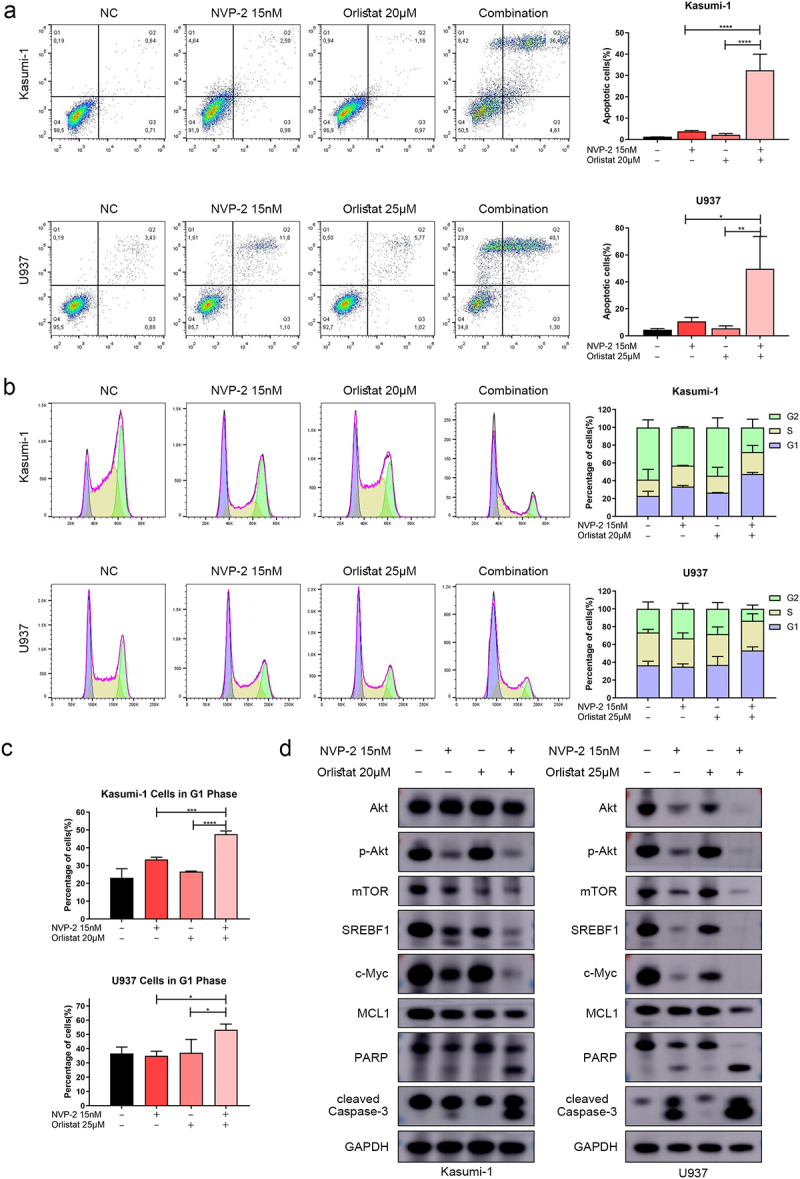
NVP-2 combined with Orlistat increased the anti-proliferative and apoptotic effects on AML cell lines. (a) Flow cytometry analyses of apoptosis in Kasumi-1 and U937 cells treated with NVP-2, Orlistat, or the combination for 16 hours. (b-c) Flow cytometry analyses of cell cycle in Kasumi-1 and U937 cells treated with NVP-2, Orlistat, or the combination for 16 hours. (d) Western blot analyses showing the expression of Akt, p-Akt, mTOR, SREBF1, c-Myc, MCL1, PARP, and cleaved Caspase-3 in Kasumi-1 and U937 cells treated with NVP-2, Orlistat, or the combination for 16 hours. GAPDH is used as the loading control. Data are shown as mean ± SD. *p < .05, **p < .01, ***p < .001, ****p < .0001.

We then proceeded to treat Kasumi-1 and U937 cells with NVP-2 and Orlistat, either in combination or alone, in order to investigate their effects and mechanisms. In these cells, the drug combination induced apoptosis along with cell cycle arrest in the G1 phase (Figure 6a-c). Through Western blot analysis, we observed decreased expressions of p-Akt, mTOR, SREBF1, c-MYC, and MCL1, as well as increased expressions of cleaved PARP and Caspase-3 in the combination group (Figure 6d). These findings suggested that treatment with NVP-2 and Orlistat effectively suppresses and kills AML cell lines. Furthermore, MCL1, c-Myc, Akt/mTOR/SREBF1 signaling pathway may play crucial roles in these processes.

## Discussion

AML is a hematopoietic malignancy with a bad prognosis. Plenty of researches have been conducted in recent years to investigate the pathogenesis and therapy of AML, aiming to identify novel prospective clinical targets and medicines with greater specificity and fewer side effects.^15^ The ultimate goal is to improve patients’ survival and quality of life.

Cyclin-dependent kinases (CDKs) are serine/threonine protein kinases that can be categorized into cell-cycle-related and transcriptional subfamilies based on their primary function.^16^ Except for the action in transcription elongation, CDK9 also regulates the cell cycle,^6^ modify chromatin and mRNA,^7^ repair DNA,^17^ and promote preadipocyte differentiation.^8^ Our analysis revealed no significant difference in CDK9 expression between AML cells and healthy tissues. However, higher CDK9 expression was linked to shorter survival times, indicating an unfavorable prognostic factor. Furthermore, our study indicated a universal expression of CDK9 in AML cell lines, and knockdown of CDK9 induced apoptosis and inhibited proliferation in Kasumi-1 and U937 cells. Additionally, we observed that CDK9 knockdown had an antitumor effect by inhibiting U937-Luc cell proliferation in the liver, bone marrow, and spleen in a leukemia mouse model. The results suggested that CDK9 could be a potential therapeutic target for AML.

Research has shown the importance of CDK9 in maintaining cell survival and proliferation of cells by promoting the continuous expression of MCL1 and c-Myc, the short half-life proteins, in AML cells.^18,19^ Furthermore, CDK9 inhibitors have been utilized for targeted tumor treatment and have demonstrated promising results in preclinical studies for AML treatment in recent years.^18^ However, previous research has indicated that the inhibitors for CDK9 were not highly selective. Identifying specific inhibitors that bind strongly and selectively to CDK9 without interacting with other molecular targets remains a challenge. Compounds with broader structural characteristics may inadvertently engage with normal biological pathways, potentially leading to side effects known as off-target effects, including detrimental impacts on healthy organs. NVP-2, a novel small molecule inhibitor, can effectively and selectively inhibit the activity of CDK9 by blocking the binding site of ATP.^20^ It has shown effectiveness in treating T-ALL cell lines and melanoma cell lines.^20,21^ This study illustrates how NVP-2 reduced AML cell proliferation and provides a theoretical basis for our future research.

Alvocidib (flavopiridol), a broad-spectrum CDK inhibitor, has undergone Phase I and II clinical trials. It has been shown to effectively inhibit CDK9, with a half-inhibitory concentration below 400 nmol/L in treating leukemia cell lines.^22^ When combined with mitoxantrone and cytarabine, Alvocidib demonstrated efficacy and good tolerability in patients. The complete response rate (CR rate) for secondary AML was 92% in the Phase I clinical trial, while it was 75% in the Phase II.^23,24^ In comparison, patients in the control group who received traditional treatment alone presented a lower CR rate of 45%.^25^ These three drugs demonstrated better therapeutic outcomes when used in combination. Single drug may exhibit adverse effects, such as impaired function and tissue damage, when administered at high doses or over extended periods. In most cases, the toxicity of a drug is determined by the therapeutic dose. However, there are certain drugs whose therapeutic dose approaches the toxic dose. Thus, toxicities may arise during treatment – cyclosporin being a notable example. Combination chemotherapy or small molecule inhibitors are commonly used clinically for more effective tumor treatment due to their higher efficacy, lower side effects, and reduced drug resistance. Tumor cells undergo multiple stages of growth involving cell division and proliferation. During these stages, dysregulation of the cell cycle and corresponding metabolic reprogramming contribute to the progression of carcinogenesis by gene mutations and aberrant immunity.^11^ It has been found that individualized administration based on metabolic disorder type in AML cells, such as dysregulation of glycometabolism or amino acid metabolism, could achieve greater clinical efficacy.^26^ Apart from chemotherapy, the IDH1/2 inhibitor Ivosidenib and the FLT3 inhibitor Gilteritinib have been given to some AML patients.^27,28^ Research has identified, in AML cells, lipid metabolic reprogramming leading to increased expression of fatty acid synthase (FASN)^13^ and induction of lipid molecule synthesis, including sphingolipids, free cholesterol, and monounsaturated fatty acids.^14^ These alterations provide sufficient energy, biofilms, and signaling molecules for proliferation. The enhanced synthesis or uptake of lipids results in an accumulation of lipid droplets, which activates oncogenic signaling pathways and modifies the tumor cell microenvironment.^29,30^ This process promotes disease progression in AML and contributes to resistance against therapy. A study conducted on diabetic rats demonstrated that upregulation of CDK9 expression is associated with increased PPAR expression levels as well as elevated lipoprotein lipase activity, thereby promoting fatty acid metabolism.^9^ Additional research involving bovine models, goose models, and breast cancer cells has also identified that CDK9 plays a role in enhancing lipid metabolism along with the expression of related genes.^31–33^ Our results from RNA-seq indicated that CDK9 regulated numerous genes associated with proliferation and apoptosis. The underlying mechanism may be the decreased transcriptional capability of P-TEFb (the CDK9/cyclin T complex) due to the inhibitor’s impact on CDK9. Interestingly, some of these genes, such as mTOR and SREBF1, might also participate in lipid metabolism and be regulated by FASN. Further in-depth study of specific pathways and targets is crucial for understanding the carcinogenic process of AML. It may provide potential therapeutic targets and enhance the effects of other small molecule inhibitors, thus improving the efficiency of AML therapy.

FASN is a multifunctional enzyme complex in mammals that directs the *de novo* synthesis of saturated fatty acids.^34^ It is structured as a homodimer, with each polypeptide chain consisting of seven regions from N-terminus to C-terminus: β-ketosynthase, malonyl/acyl transferase, dehydratase, enoylreductase, β-ketoreductase, acyl-carrier protein, and thioesterase.^35,36^ However, over-expression of FASN promotes tumorigenesis and progression in cancers such as prostate and breast cancer.^37,38^ Previous research has shown that reducing FASN expression accelerates the differentiation of APL cell lines and re-sensitizes ATRA refractory non-APL AML cells.^13^ Orlistat, a small molecule inhibitor, inhibits the activity of FASN by binding to its thioesterase.^39^ It also decreases saturated fatty acid production and affects the function of related lipid metabolism genes to inhibit tumor cell proliferation.^39,40^ It is shown that Orlistat dose-dependently reduced Kasumi-1 and U937 cell viability. Furthermore, it was demonstrated that the combination of NVP-2 and Orlistat, in these cells, enhanced anti-proliferative and apoptotic effects.

Apoptosis is a form of programmed cell death,^41^ in which the immune system and multiple apoptosis-related proteins play crucial roles.^42^ Gene mutations can result in the up-regulated expression of BCL-2 family proteins, c-Myc, and MEIS1 in myeloid progenitor cells. This inhibits apoptosis and helps with the progression of leukemia.^42,43^ On the surface of AML cells, FasL over-expression can induce immune disorder, reduce T cell cytotoxicity,^44^ and protect AML cells from immune attack. Reactivating the apoptotic pathway shows promise as a treatment for AML.^45^ Down-regulation of c-Myc after CDK9 inhibition also reduces MCL1 expression in apoptosis early stages.^46^ Activation of BCL-2 family members Bax, Bak, and Bim upon loss of MCL1 sequestering function alters mitochondrial membrane permeability and leads to cytochrome c release.^47–49^ Apaf-1 forms apoptosomes with cytochrome c, causing self-polymerization and then recruitment of Caspase-9.^50^ Subsequently, its activation triggers caspase-3 activation, PARP cleavage, and ultimately apoptosis.^51^ Mitochondria not only play a core position in the apoptotic route, but also serve as sites for fatty acid oxidation.^52^ According to Samudio’s research, inhibiting fatty acid synthesis or oxidation enhances the apoptotic effect by disrupting mitochondrial membrane integrity through reduced fatty acid transmembrane transport and oxidation.^53^

Infinite proliferation is a key characteristic of malignant cells, and anti-tumor therapy aims to inhibit tumor growth. Tumor cell proliferation is driven by cell cycle cycling. Thus, the observed AML cell suppression in the study may be attributed to cell cycle arrest. Activation of certain signaling pathways and over-expression of specific genes promote tumor cell proliferation. For example, CDK9 and MNK enhance AML cell survival and proliferation through phosphorylation of eIF4E and activation of the PI3K/Akt/mTOR pathway.^54–56^ Additionally, research indicated that that FASN promotes tumor proliferation and invasion, such as gastrointestinal stromal tumor and liver cancer, by activating the Akt/mTOR pathway.^57,58^ Furthermore, SREBF1 is directly regulated by the PI3K/Akt/mTOR pathway.^59^ Our findings also indicate that CDK9 and FASN can regulate the Akt/mTOR/SREBF1 pathway in Kasumi-1 and U937 cells. Research has shown that inhibiting c-Myc and blocking the Akt/mTOR pathway can induce a G1 phase cell cycle arrest because of secondary down-regulation of Cyclin D1 and dysregulation of the cell cycle.^60,61^ In short, phosphorylation of retinoblastoma protein induced by Cyclin D1 binding to CDK4/6 results in loss of its suppression function on E2F transcription factor, causing expression of several G1- to S-phase associated proteins and promoting cells to enter S phase. Cyclin D1 down-regulating can leads to a G1 phase cell cycle arrest.^62^

In conclusion, this study investigated the functions of CDK9 in AML cells through *in vivo* and *in vitro* experiments. The research initially discovered that NVP-2 impacted the transcription of numerous genes, such as SREBF1, in U937 cells. Furthermore, the combined treatment of NVP-2 and Orlistat could reduce AML cell proliferation and induce apoptosis. It is suggested that MCL1, c-Myc, and Akt/mTOR/SREBF1 may be critical factors and pathways following the treatment. However, it should be noted that this study did not examine the therapeutic effects or metabolic time of NVP-2 and Orlistat in a leukemia mouse model, and the side effects and toxic effects of these drugs have yet to be assessed. This report on the combination of NVP-2 and Orlistat for treating AML cell lines is groundbreaking and may have potential as a clinical candidate for treating patients with AML.

## Materials and methods

### Cell lines and culture

Human AML cell lines, including Kasumi-1, NB4, U937, THP-1, and K562, were obtained from the cell bank of the American Type Culture Collection and cultured in RPMI-1640 medium (XP Biomed Ltd, Shanghai, China) containing 10% fetal bovine serum (XP Biomed Ltd, Shanghai, China), and 1% penicillin – streptomycin (Beyotime Biotechnology, Shanghai, China). 293 FT cell was obtained from the cell bank of the Chinese Academy of Science and cultured in DMEM medium (XP Biomed Ltd, Shanghai, China) containing 10% fetal bovine serum (Shuangru Biotechnology, Shanghai, China), and 1% penicillin – streptomycin (Beyotime Biotechnology, Shanghai, China). All cells were cultured in a humidified incubator with 5% CO2 at 37°C and confirmed by short tandem replicates between 2022 and 2023.

### Preparation and infection of lentivirus

Short hairpin RNA (shRNA) targeting CDK9 were constructed using pLKO.1-puro lentivirus (IGE Biotechnology LTD, Guangzhou, China) as a vector. The Sequence used here are listed in Table S1. Purified plasmids and packaging plasmids, including psPAX2 (Addgene, MA, USA) and pMD2G (Addgene, MA, USA), were transfected into 293 FT cells using PEI (Polysciences, PA, USA) according to the manufacturer’s instructions. The supernatant with lentivirus was collected after 48 hours and filtered through a 0.45 μm filter. AML cells were transfected with lentivirus while adding 10 μg/mL polybrene (Beyotime Biotechnology, Shanghai, China) for 24 hours. Stable cell lines were selected with 2ug/ml puromycin (Thermo, USA).

### Cell proliferation and viability measurement

After transfection, AML cell lines were seeded in 96-well plates with 2 × 10^3 cells per well. CCK-8 (Bimake, USA) and a spectrometer were used for measuring the cell viability. The OD value was measured at 450 nm. Cell density was measured three times and repeated in at least three separate trials.

### Cytotoxicity assay

AML cell lines were seeded in 96-well plates with 3 × 10^4 cells per well. Cells were treated with gradient concentrations of NVP-2 (from 1.95 nM to 500 nM) (MedChemExpress, USA) for 24 hours, or gradient concentrations of Orlistat (from 2.5 μM to 40 μM) (MedChemExpress, USA) for 48 hours, or 10 μM Orlistat combined with gradient concentrations of NVP-2 (from 1.95 nM to 500 nM) for 24 hours, while control group added an equal volume of DMSO (<0.1% volume) (Sigma-Aldrich, USA). CCK-8 and a spectrometer were used to measure the cell viability. The OD value was measured at 450 nm. Cell density was measured three times and repeated in at least three separate trials.

### RNA-seq and data processing

RNA-seq assay was performed following the Novogene (Beijing, China) protocol. U937 cells were treated with 20 nM NVP-2 or the same volume DMSO for 12 hours, then the differentially expressed genes were determined via RNA-seq. Total RNA was isolated using Trizol (Invitrogen, USA) reagent. Novogene performed RNA purification, library construction, and sequencing. The raw reads were filtered and clean reads were mapped by HISAT. DESeq2 analysis was used to identified the differentially expressed genes (log2Foldchange < −1 or > 1, *p* < .05).

### Quantitative real-time PCR

U937 cells were transfected with lentivirus, and stable cell lines were selected with 2ug/ml puromycin (Thermo, USA). According to the manufacturer’s protocol, total RNA was isolated using TRIZOL reagent (Invitrogen, USA) and then transcribed into cDNA by the High-Capacity cDNA Reverse Transcription Kit (Applied Biosystems, USA). Quantitative Real-time PCR was performed using the LightCycler® 480 (Roche, Germany). The primers used here are listed in Table S2. GAPDH expression was used as the internal reference.

### Western blot analysis

Cells were collected after treatment, and total protein was obtained with RIPA lysis buffer (Beyotime Biotechnology, Shanghai, China). Western blot was performed, and PVDF membranes were incubated overnight at 4°C using the following primary antibody, including GAPDH (AP0063, Bioworld Technology, USA), CDK9 (2316, CST, USA), FASN (3180, CST, USA), c-Myc (13987, CST, USA), PARP (9532, CST, USA), cleaved Caspase-3 (9661, CST, USA), Akt (4685, CST, USA), Phospho-Akt (4060, CST, USA), mTOR (2983, CST, USA), MCL1 (ab32087, Abcam, UK), SREBF1 (ab28481, Abcam, UK).The next day, the membranes were incubated with goat anti-rabbit IgG (H+L) (111-035-003, Jackson, USA) and goat anti-mouse IgG (H+L) (115-035-003, Jackson, USA) secondary antibodies for 1 hour at room temperature. With ECL ultra-sensitive luminescent solution (Applygen Technologies, Beijing, China) added to the membranes, visualization and analysis was performed with LAS 4010 imaging system (GE Healthcare Life Sciences, UK). GAPDH was used as the internal control.

### Soft agar assay

Kasumi-1 cells were treated with 15 nM of NVP-2 and 20 μM of Orlistat for 11 days, combined or alone. U937 cells were treated with 15 nM of NVP-2 and 25 μM of Orlistat for 14 days, combined or alone. Treated cells were fixed overnight in 4% paraformaldehyde fixative (Beyotime Biotechnology, Shanghai, China) and stained with Giemsa solution (Beyotime Biotechnology, Shanghai, China), and then the clones were counted.

### Apoptosis analysis

Kasumi-1 cells were treated with 15 nM of NVP-2 and 20 μM of Orlistat for 16 hours, combined or alone. U937 cells were treated with 15 nM of NVP-2 and 25 μM of Orlistat for 16 hours, combined or alone. Cells were collected, washed with cold PBS, suspended in a binding buffer (BD Biosciences, USA), and stained with FITC-Annexin V (BD Biosciences, USA) and PI solution (BD Biosciences, USA) according to the manufacturer’s protocol. Apoptosis was analyzed by flow cytometry (Beckman, USA).

### Cell cycle analysis

Kasumi-1 cells were treated with 15 nM of NVP-2 and 20 μM of Orlistat for 16 hours, combined or alone. U937 cells were treated with 15 nM of NVP-2 and 25 μM of Orlistat for 16 hours, combined or alone. Cells were fixed by using 70% ethanol at 4°C overnight and punched with 0.5% TritonX-100. Then cells were treated with RNase A (CST, USA), stained with PI (Sigma-Aldrich, USA), and analyzed by flow cytometry (Beckman, USA).

### *In vivo* experiments

Animals were used following a protocol reviewed and approved by the Animal Care and Use Committee at Children’s Hospital of Soochow University. M-NSG mice (Cat. NO. NM-NSG-001) were purchased from Shanghai Model Organisms Center, Inc. U937 cells expressing firefly luciferase (U937-Luc) were transfected with sh-NC or sh-CDK9. Twenty M-NSG mice (4–6 weeks old) were randomly divided into sh-NC group and sh-CDK9 group and then injected with 1 × 10^6 cells per mouse via the tail vein, with an equal number of mice in each group. Bioluminescence imaging using the BERTHOLD (Germany) *in vivo* imaging system was conducted on Day 6, 9, and 12 after mice were injected. To euthanize mice. Mice liver, spleen, and bone marrow were collected for HE staining and IHC staining to facilitate further microscopic examination.

### Data statistics and analysis

All experiments were independently performed in triplicate at least three times. Statistical analysis was performed with GraphPad Prism 7.00 (GraphPad Software, USA). The *t*-test was used for comparison between two groups, one-way ANOVA was used for comparison between multiple groups, and Tukey’s method was used for multiple comparisons after ANOVA. The *p* values with statistical significance are indicated as * *p* < .05, ** *p* < .01, *** *p* < .001, **** *p* < .0001, and ns not significant.

## Supplementary Material

Supplemental Material

TableS2.docx

FigureS2.tif

TableS3.docx

TableS1.docx

FigureS1.tif

## Data Availability

All data generated or analyzed during this study are included in this article. The datasets used and/or analyzed during the current study are available from the corresponding authors on reasonable requests.
