# Supplementary material for: NVP-2, in combination with Orlistat, represents a promising therapeutic strategy for acute myeloid leukemia
Source: Cancer Biol Ther. 2025 Jan 12;26(1):2450859. doi: 10.1080/15384047.2025.2450859 (PMC11730633; doi:10.1080/15384047.2025.2450859)
Supplement: Supplemental Material [file KCBT_A_2450859_SM9722.docx]

**Supplementary Figure caption**

**Figure S1** CDK9 knockdown inhibited the proliferation of U937-Luc cells in the spleen and bone marrow. Representative images of mice spleen (a) and bone marrow (b) by HE staining or IHC staining (Ki67).

**Figure S2** Orlistat enhanced NVP-2 inhibitory effect in the treatment of AML cell lines *in vitro*. (a) Typical images of cell colony in Kasumi-1 and U937 cells treated with NVP-2, Orlistat, or the combination as observed by microscope.
