# Supplementary material for: NVP-2, in combination with Orlistat, represents a promising therapeutic strategy for acute myeloid leukemia
Source: Cancer Biol Ther. 2025 Jan 12;26(1):2450859. doi: 10.1080/15384047.2025.2450859 (PMC11730633; doi:10.1080/15384047.2025.2450859)
Supplement: TableS2.docx [file KCBT_A_2450859_SM0110.docx]

**Supplementary Table 2** Primer sequences used in RT-PCR

| **Target** | **Primer sequences (5′ to 3′)** | |
| --- | --- | --- |
| CDK9 | Forward | ATGGCAAAGCAGTACGACTCG |
|  | Reverse | GCAAGGCTGTAATGGGGAAC |
| c-Myc | Forward | GGCTCCTGGCAAAAGGTCA |
|  | Reverse | CTGCGTAGTTGTGCTGATGT |
| Myb | Forward | AAGGTCGAACAGGAAGGTTATC |
|  | Reverse | ACTGTTCTTCTGGAAGCTTGT |
| SREBF1 | Forward | ACGCCGCCTCCTTCCACCAT |
|  | Reverse | AAGGCAGGCACCGACGGGTA |
| mTOR | Forward | ATGCAGCTGTCCTGGTTCTC |
|  | Reverse | AATCAGACAGGCACGAAG |
| CDK6 | Forward | GGATAAAGTTCCAGAGCCTGGAG |
|  | Reverse | GCGATGCACTACTCGGTGTGAA |
| GAPDH | Forward | TGCACCACCAACTGCTTAG |
|  | Reverse | GATGCAGGGATGATGTTC |

Note: CDK9, cyclin-dependent kinase 9; SREBF1, sterol regulatory element-binding factor 1; mTOR, mammalian target of rapamycin; CDK6, cyclin-dependent kinase 6; GAPDH, glyceraldehyde-3-phosphate dehydrogenase
