# Supplementary material for: NVP-2, in combination with Orlistat, represents a promising therapeutic strategy for acute myeloid leukemia
Source: Cancer Biol Ther. 2025 Jan 12;26(1):2450859. doi: 10.1080/15384047.2025.2450859 (PMC11730633; doi:10.1080/15384047.2025.2450859)
Supplement: TableS3.docx [file KCBT_A_2450859_SM0108.docx]

**Supplementary Table 3** The values of Figure 4d results

| **Gene Name** | **Group** | | | |
| --- | --- | --- | --- | --- |
|  | **NC_1** | **NC_2** | **CDK9i_1** | **CDK9i_2** |
| Myc | 0.929644329 | 0.79991069 | -0.85419381 | -0.875361208 |
| Myb | 0.697753239 | 1.01930839 | -0.859033731 | -0.858027898 |
| MCL1 | 0.77265623 | 0.949983391 | -0.952944797 | -0.769694823 |
| Bcl-2 | 0.820190513 | 0.908543253 | -0.925915487 | -0.80281828 |
| Caspase-3 | 0.57962652 | 1.111035507 | -0.830096919 | -0.860565108 |
| Caspase-9 | 0.851453122 | 0.880130419 | -0.841223051 | -0.89036049 |
| FASN | 1.033001603 | 0.680808077 | -0.877352834 | -0.836456846 |
| Akt1 | 1.12634552 | 0.551837571 | -0.935714058 | -0.742469033 |
| mTOR | 0.732539263 | 0.98985186 | -0.872683238 | -0.849707885 |
| SREBF1 | 1.0905896 | 0.607160242 | -0.868793541 | -0.828956301 |
| Cyclin D1 | -0.053128754 | 1.355042641 | -1.046903211 | -0.255010676 |
| CDK6 | 0.589698357 | 1.10377769 | -0.8469599 | -0.846516147 |
| IDH1 | 0.684896709 | 1.029283293 | -0.823282755 | -0.890897247 |
| FLT3 | 0.573645166 | 1.114526089 | -0.800486739 | -0.887684517 |
| MEIS1 | 0.658151729 | 1.051426039 | -0.852389605 | -0.857188163 |
| E2F1 | 1.108347227 | 0.58321805 | -0.864842906 | -0.826722371 |

Note: MCL1, Myeloid cell leukemia 1; Bcl-2, B cell lymphoma/leukemia-2; FASN, Fatty acid synthase; mTOR, mammalian target of rapamycin; SREBF1, sterol regulatory element-binding factor 1; CDK6, cyclin-dependent kinase 6; IDH1, Isocitrate dehydrogenase 1; FLT3, Fms-like tyrosine kinase 3; MEIS1, Myeloid ecotropic viral integration site 1; E2F1, Early 2 Factor 1

Differences between genes in raw data have been normalized.
