# Supplementary material for: NVP-2, in combination with Orlistat, represents a promising therapeutic strategy for acute myeloid leukemia
Source: Cancer Biol Ther. 2025 Jan 12;26(1):2450859. doi: 10.1080/15384047.2025.2450859 (PMC11730633; doi:10.1080/15384047.2025.2450859)
Supplement: TableS1.docx [file KCBT_A_2450859_SM0107.docx]

**Supplementary Table 1** shRNA used to knockdown CDK9

| **Name** | **Sequence (5′ to 3′)** |
| --- | --- |
| Homo-CDK9-sh1 | CCGGGCTGCAAGGGTAGTATATACCCTCGAGGGTATATACTACCCTTGCAGCTTTTTGAATT |
| Homo-CDK9-sh2 | CCGGGGTGATGCAGATGCTGCTTAACTCGAGTTAAGCAGCATCTGCATCACCTTTTTGAATT |
| Homo-CDK9-sh3 | CCGGGCCTATGTGCGTGACCCATACCTCGAGGTATGGGTCACGCACATAGGCTTTTTGAATT |

Note: CDK9, cyclin-dependent kinase 9
